# Generation of a Broadly Cross-Neutralizing Antibody Fragment against Several Mexican Scorpion Venoms

**DOI:** 10.3390/toxins11010032

**Published:** 2019-01-10

**Authors:** Lidia Riaño-Umbarila, Ilse V. Gómez-Ramírez, Luis M. Ledezma-Candanoza, Timoteo Olamendi-Portugal, Everardo Remi Rodríguez-Rodríguez, Guillermo Fernández-Taboada, Lourival D. Possani, Baltazar Becerril

**Affiliations:** 1 CONACYT, Instituto de Biotecnología—Universidad Nacional Autónoma de México, Apartado Postal 510-3, Cuernavaca, Morelos 62250, Mexico; umbarila@ibt.unam.mx; 2 Departamento de Medicina Molecular y Bioprocesos, Instituto de Biotecnología, UNAM, Apartado Postal 510-3, Cuernavaca, Morelos 62250, Mexico; ilsegmz@ibt.unam.mx (I.V.G.-R.); lmlecan832@gmail.com (L.M.L.-C.); timoteo@ibt.unam.mx (T.O.-P.); everardoremi@gmail.com (E.R.R.-R.); gft@ibt.unam.mx (G.F.-T.); possani@ibt.unam.mx (L.D.P.)

**Keywords:** cross-neutralization, directed evolution, phage display, polyvalent antivenom, scFv, scorpion

## Abstract

The recombinant antibody fragments generated against the toxic components of scorpion venoms are considered a promising alternative for obtaining new antivenoms for therapy. Using directed evolution and site-directed mutagenesis, it was possible to generate a human single-chain antibody fragment with a broad cross-reactivity that retained recognition for its original antigen. This variant is the first antibody fragment that neutralizes the effect of an estimated 13 neurotoxins present in the venom of nine species of Mexican scorpions. This single antibody fragment showed the properties of a polyvalent antivenom. These results represent a significant advance in the development of new antivenoms against scorpion stings, since the number of components would be minimized due to their broad cross-neutralization capacity, while at the same time bypassing animal immunization.

## 1. Introduction

In Mexico, scorpion stings represent an important public health problem due to the existence of a great diversity of medically important species. Until recently, only eight species of scorpions of the genus *Centruroides* had been considered dangerous to humans. We have confirmed the toxicity of a total of 14 species of scorpions [1] (six of them formally unknown as dangerous to humans). This means that the complexity of the problem is much greater than previously thought, since there is a significant number of toxic species that inhabit 15 states located on the Pacific Ocean boundary and central parts of the country. The deep characterization of the venom of some of these species enabled the identification of toxic components that correspond to a few peptides of only 66 aa [2,3]. These peptides are defined as neurotoxins, because they act on the sodium channels of excitable cells modulating their gate function, and thus alter the transmission of nerve impulses and end up triggering serious symptoms of intoxication such as asphyxia, partial paralysis, and cardiopulmonary shock. The toxins that have been identified so far share a high level of identity in their primary structure (around 78%) (Table 1) and the same fold (one α helix and three β antiparallel strands), but show differences in the stability, toxicity, and biological activity on human sodium channels (reviewed in [4,5]). It has been shown that few differences in the sequence modify the recognition of their targets; for example, Cn2 (the main toxin of the scorpion *C. noxius*) only affects the human sodium channel 1.6 [6], while the toxins of *C. limpidus* (Cll1 and Cll2, harboring 10 and nine changes with respect to Cn2) affect most of the sodium channels [7] and Cl13 (showing 15 changes respect to Cn2) affects human sodium channels 1.4, 1.5, and 1.6 [8].

In general, the production of antivenoms through the immunization of horses has helped to save many lives. However, it still has several drawbacks such as a limited therapeutic efficiency, a lack of homogeneity attributable to the variability of the animal immune response, the possibility of secondary reactions due to its heterologous origin, as well as a relatively high cost of production, reviewed in [9]. In Mexico, even though the venoms of only four species of scorpions are used for the production of commercial antivenom, these protect against several toxic species [4,5]; however, the exact number of scorpion venoms that are neutralized has not yet been determined. The polyclonal antibodies obtained from this process recognize all of the venom components, including toxins, which typically correspond to less than 10% of the whole venom [10,11,12]. The polyvalent character of scorpion antivenom is explained by the ability to cross-neutralize the venom of different species, which is associated with the sequence similarity of the toxic components.

Numerous examples of well-characterized single antibodies with a broad cross-reactivity and the capability of neutralizing various targets are currently known, particularly those that were raised up against viruses [13,14,15,16,17,18]. In contrast, the evaluation of the cross-reactivity of polyclonal antivenoms is complicated due to the wide diversity of their components. Fortunately, there are new methodologies to make a better characterization of such cross-reactivity, which were reviewed in [19]. The generation of recombinant antibody fragments against scorpion toxins enables the study of their cross-reactivity properties. In previous works, two parental antibodies of human origin in a single-chain antibody fragment (scFv) format named C1 and 3F, which is specific against the Cn2 toxin, were isolated. It was determined that they recognize different epitopes in the toxin. These scFvs were evolved (in vitro matured), obtaining a set of variants that showed a high neutralizing capacity but limited cross-reactivity [20,21,22]. In this work, we present the final stages of maturation of scFv C1, which allowed the generation of some variants with a broader cross-reactivity. Initially, scFv RU1 was generated [23], which was further matured, giving rise to scFv ER-1 [22]. This latter was the parental scFv from which broad cross-reacting variants were obtained by means of different strategies. scFv 10FG2 showed the broadest cross-reactivity, which was capable of neutralizing an estimated 13 scorpion toxins and the fresh venom of five Mexican scorpions.

## 2. Results

### 2.1. In Vitro Maturation of Antibody Fragments

**Figure 1:**
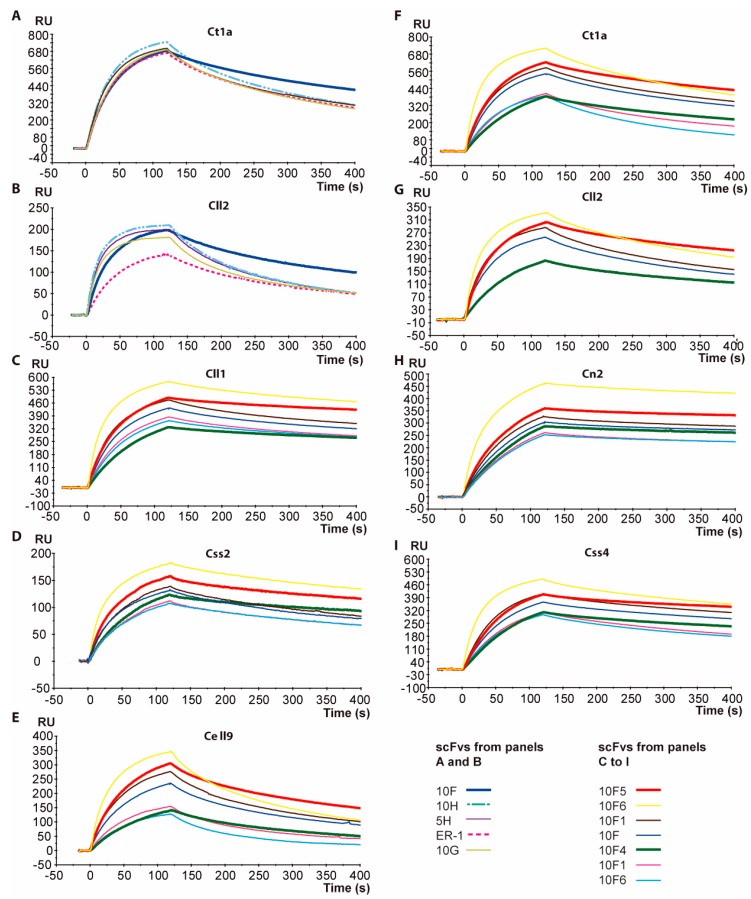
Comparisons of Biacore sensorgrams which depict the interactions between recombinant antibody fragments and immobilized toxins. The association (first 120 s) and dissociation (121–400 s) phases of the sensorgrams are shown. All of the evaluations were performed with the pure monomeric proteins of the different scFvs at a concentration of 100 nM and with the respective toxins previously immobilized on CM5 chips. Panels (**A**,**B**), variants of the single-chain antibody fragment (scFv) ER-1 selected during the maturation toward the Cll2 toxin after the second and third rounds of biopanning. The scFv 10F is highlighted. The panels (**C** to **I**) correspond to the evaluation of the corresponding 10F scFv variants. The variants 10F4 and 10F5 are highlighted by means of thick sensorgrams.

scFv ER-1 [22], a variant derived from scFv C1 (Table 2) which showed a good level of recognition against toxin Cll2 (the most abundant toxic component of *C. limpidus* venom) [8] and Ct1a (the main toxin of *C. tecomanus* venom) [24] (Table 1), was modified in order to improve its recognition toward these toxins. As a first step, a library of variants of scFv ER-1, which was generated by the saturation mutagenesis of residues 235 and 236 located in the CDR3 of the light chain, was constructed. These positions were selected based on structural analyses that revealed some contacts with amino acids located at the carboxy terminus of the toxins. Following the methods described in the experimental procedures section, a library of 4 × 10^5^ transformants was obtained. The variability within the library was confirmed by sequencing 10 random clones, which presented different mutations at these selected positions. After two independent rounds of biopanning against Cll2 and Ct1a toxins, scFv 10F was isolated (Table 2). This variant showed an improved binding toward both toxins. The analysis of the sequence of this variant indicated that the changes that helped improve the interaction with both toxins were L235T and I236L. A third round of screening was carried out, and all of the selected variants were assessed by means of surface plasmon resonance (SPR) in a sensor of molecular interactions in real time (BiacoreX, GE Healthcare, Upsala, Sweden). The comparison of the respective Biacore sensorgrams for the interaction between scFvs and toxins indicated that none of the generated variants showed any improvement as compared to scFv 10F, which was selected in the second round of biopanning (Figure 1, panels A and B).

Encouraged by this improved cross-reactivity shown by scFv 10F in terms of binding strength to the toxins, we decided to evaluate the effect of combining other mutations such as 107A/S, 110N/T, and 164 S/I (Table 2), which occurred during the previous directed evolution processes performed on this scFv’s family [22]. Additionally, an analysis of the conserved residues within the frameworks (FW) lead to the hypothesis that residue A204 should be changed to G204, since it is a conserved residue within germlines. Six variants with the combinations that were shown in Table 2 were generated. Their assessment in terms of recognition levels in Biacore showed the significant improvements of some of them against several toxins (Figure 1, Panels C to I). The sensorgrams that are shown depict the interaction levels of six scFv 10F variants against seven different scorpion toxins. These results clearly indicate that variants 10F2 and 10F3 showed a lower level of interaction toward the respective toxins compared to scFv 10F, while the 10F1 variant showed a barely higher recognition. On the other hand, scFv 10F6 showed the highest level of association against several toxins, whereas in the dissociation phase, a greater tendency to dissociate from toxins can be seen, except from Cn2. This behavior contrasts with scFvs 10F4 and 10F5, whose sequences differ only at position 110 (scFv 10F4: N and scFv 10F5: T). We have observed that the presence of T instead of N at position 110 (Table 2) propitiates greater association kinetics with the toxins. In contrast, in the dissociation phase, a similar kinetics was observed when comparing the two corresponding sensorgrams (Figure 1). It was also notorious that the interactions of these two scFvs were similar against all of the evaluated toxins. However, the comparative analysis showed that variant 10F5 (S107, T100, S164 and G204), which is now called scFv 10FG2, was the best ranked when interacting with the seven toxins that were used for these evaluations.

### 2.2. Affinity Determination of scFv 10FG2

Using a sensor of molecular interactions in real time (Biacore X), the kinetic constants of the association and dissociation of scFv 10FG2 toward six of the most abundant and toxic components of scorpion venoms were determined (Table 3). The values of the association constants were similar, ranging from one to three × 10^5^ M^−1^s^−1^, whereas the values of the corresponding dissociation constants exhibited significant variations (6.3 to 100 × 10^−5^ s^−1^). These differences in the kinetic constants of 10FG2 with toxins were reflected in the constant of dissociation in the equilibrium or affinity constant (*K_D_*) being the highest toward the Cll1 toxin, followed by the Cn2, Css2, Cll2, Cell9, and Ct1a toxins, respectively. On the other hand, when comparing the *K_D_* values of the scFv ER-1 determined against four toxins previously evaluated and compared to the ones corresponding to scFv 10FG2, increases of 5.6 times for Cll1, 1.4 times for Cn2 and Cll2, and 3.6 times for Ct1a were observed. Due to the increase in the affinity of scFv 10FG2 for toxins, different neutralization challenges were carried out.

### 2.3. Toxin Neutralization Assays

In these assays, we evaluated the ability of scFv 10FG2 to neutralize four different toxins (Cll1, Cll2, Cell9, and Ct1a). A lethal dose 100 (LD_100_) was injected to the mice in the control groups. For each experimental group, the same corresponding amount of toxin was mixed with scFv 10FG2, using a one to five molar ratio of toxin:scFv (Table 3). The administration of each mixture resulted in 100% survival with no symptoms of intoxication in contrast to the control groups, where all of the mice died except for Cll1, where one mouse survived. Since all of the tested toxins were neutralized (Table 1), a preliminary neutralization assay was carried out with the whole venoms of the scorpions *C. limpidus, C. elegans,* and *C. tecomanus.* The scFv 10FG2 showed a protection of 80% to 90% using one LD_50_ of venom (data not shown). Envenoming symptoms were observed during the experiment, indicating that scFv 10FG2 did not eliminate the effects of the whole venom of these three species. Subsequently, the neutralization capacity of other available venoms was evaluated.

### 2.4. Venom Neutralization and Rescue Assays

scFv 10FG2 was also evaluated against another six Mexican scorpion venoms using different LD_50_ values of venom while fixing the amount of scFv corresponding to the one needed to neutralize one LD_50_ of venom. The amount of scFv was determined by assuming that the toxins represent 10% of the total venom, and that optimal neutralization occurs when the toxin:scFv molar ratio is 1:10 (Table 4). The mixture of venom and scFv was administered intraperitoneally after incubation for 30 min at 37 °C. This is a very demanding condition, considering that scFv 10FG2 is the only scFv that was employed, compared to the commercial antivenom, which contains multiple antibodies against several toxins. For the six species tested (*C. hirsutipalpus*, *C. infamatus*, *C. species nova* (sp nov) “B” (from Sonora), *C. noxius, C. suffusus*, and *C.* sp nov “A” (from Oaxaca and a not yet described species), we found that scFv 10FG2 was able to neutralize one LD_50_ of all of the venoms with 100% effectiveness at a 1:10 molar ratio. However, in the case of *C.* sp nov. “A”, some symptoms of intoxication were observed. When the amount of venom was increased to two and three LD_50_ (the molar ratio toxin:scFv ratio was reduced to 1:5 and 1:3.3, respectively), five out of six species were fully protected. For *C.* sp. nov. “B”, five out of six mice survived when using a molar ratio of 1:3.3. When the molar ratio was diminished to 1:2.5, the toxic effects of three venoms were inhibited (Table 4), and finally, when a molar ratio of 1:2 was assessed, only the venom of *C. infamatus* was totally neutralized. Based on these results, we decided to perform rescue assays in order to evaluate the neutralizing capacity of scFv 10FG2 in conditions of an acute envenoming caused by five out of the six venoms. The assay with venom of *C.* sp nov “A” was discarded because scFv 10FG2 was not capable of 100% neutralizing this venom.

In order to simulate an accident by scorpion sting, mice were injected with three LD_50_ of each venom, and after a lapse of five to 10 min, the estimated amount of scFv that was necessary to neutralize the toxins was administered intraperitoneally. Assuming that toxins correspond to 10% of the venom, a molar ratio of 1:10 (toxin:antibody) was injected. The results of these rescue assays are shown in Table 4, where a 100% protection of the mice was observed for the venoms of *C. hirsutipalpus*, *C. infamatus*, and *C.* sp nov “B”. Neutralization of the venoms of *C. noxius* and *C. suffusus*, which are the most toxic in Mexico, required higher amounts of scFv. When the amount of antibody was increased to a molar ratio of 1:20, the protection was 100%.

### 2.5. Structural Analysis of the Interaction of scFv 10FG2 with Toxins

**Figure 2:**
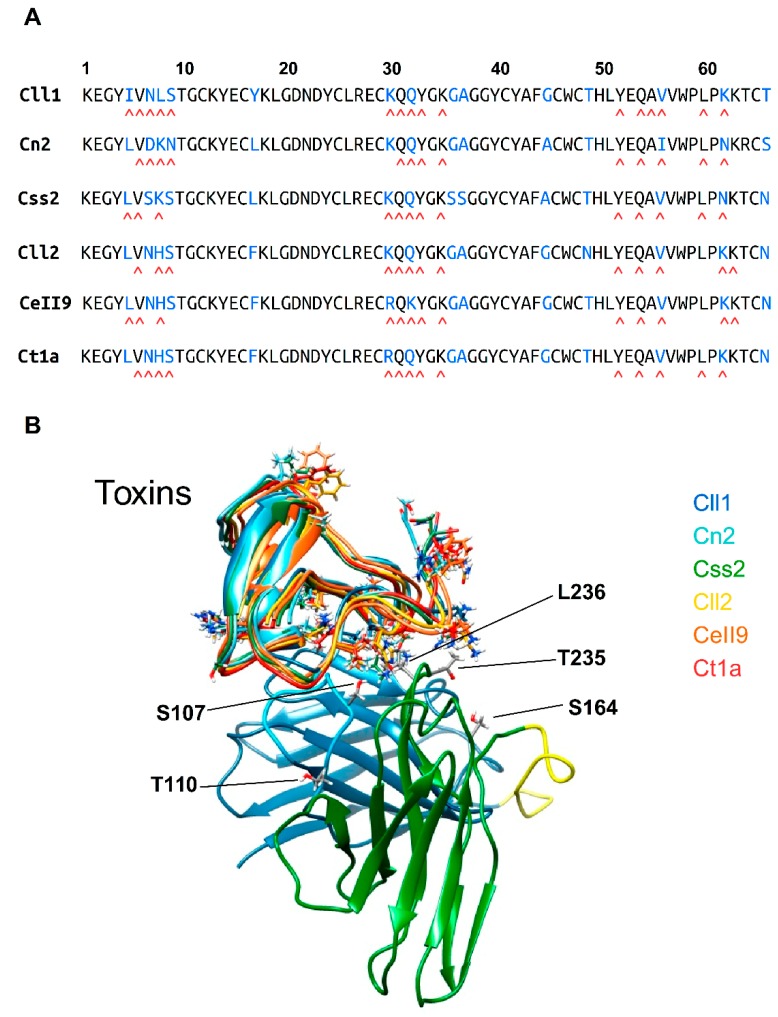
Interaction analysis between scFv 10FG2 and toxins. (**A**) Sequence alignment of the toxins. Below each sequence, the residues that interact with scFv 10FG2 are indicated by a red caret symbol. (**B)** Structural alignment of the six toxins analyzed. Toxin names are shown at the right top of the panel in different colors. The regions with residues that are different among the six toxins are indicated by the respective side chains of the corresponding residues. The lower side of the panel shows the scFv 10FG2. The V_H_ domain is colored in blue, the V_L_ domain is colored in green, and the linker sequence that joins the V_H_ and V_L_ domains is colored in yellow. The amino acid residues in scFv 10FG2 that are considered important for toxin binding are indicated with a single letter code.

Using the crystallographic structure data of the ternary complex scFv LR-Cn2-scFv RU1 [23] as the template, the models of scFv 10FG2 with the toxins evaluated in this work (Cn2, Cll1, Cll2, Css2, Ct1a, and Cell9) were constructed following the procedures described in the experimental procedures section. The alignment of the primary structure and the existing identity between them are shown in Figure 2A. The high percentage of identity that the toxins have with respect to the Cn2 toxin (Cll1: 84%, Css2: 89%, Cll2: 84%, CeII9: 83%, Ctla: 84% and Cl13: 77%) enabled modeling the complexes. Figure 2B shows the over-position of the three-dimensional (3D) structures of the six models generated in which the characteristic folding of the toxins affecting the sodium channels was observed [26,27]. The analyses of the corresponding simulations of molecular dynamics (MD) of the scFv 10FG2 with each toxin enabled studying the interactions in each complex in detail. The number and type of contacts are summarized in Table 5, where it is possible to observe that most of the contacts of scFv 10FG2 are similar among all of the toxins.

## 3. Discussion

The expansion of the neutralizing capacity of the scFv family of variants here reported represented a great challenge, since previous attempts were not sufficiently effective. In this work, we show the final stages of the in vitro maturation of the scFv ER-1 [22], which despite recognizing several toxins, was capable of neutralizing only a few of them. To extend the scFv ER-1 neutralization capacity, a semi-rational strategy was first conducted, taking advantage of the structural data from the crystallographic ternary complex LR-Cn2-RU1. Positions 235 and 236 located at the interface of the toxin and the scFv were mutagenized to saturation in the ER-1 sequence, and the library was screened against toxin Cll2 and Ct1a. After evaluating the selected variants of the second and third biopanning rounds against toxins, we observed that M, V, T, and S residues were selected for position 235, while for position 236, they corresponded to I, F, V, M, and W. These results, as well as the ones that emerged from MD simulations where T235 and L236 positions were evaluated, indicated that an important set of interactions was established with a hydrophobic patch formed by residues V56, L60, and L8 in the toxin (Table 5). The presence of conserved residues at these positions explains the cross-reactivity shown by scFv 10F with toxins Cll2, Ct1a, Cn2, and Cll1. Additionally, the improved binding strength that was observed in this evolved scFv (Figure 1A,B) indicated that this set of interactions represent an important component of the total network of interactions between scFv 10F and the evaluated toxins.

These results prompted us to evaluate the effect of combining the mutations A/S 107, N/T110, S/I164 (Table 2), and G 204 using scFv 10F as the template by site-directed mutagenesis. The set of generated variants were evaluated by SPR binding assays (Biacore) (Figure 1, panels C to I). The best combination of mutations was present in the scFv called 10FG2 (S107, T110, S164, and G204). This scFv showed the highest and the lowest association and dissociation constants with these toxins, respectively. The *K_D_* values reflected that the interaction of scFv 10FG2 with the toxins was variable. However, a key parameter to assess the relative importance of these differences was the dissociation constant. The re-conversion of this parameter enables the calculation of the “residence time” (T_R_) (Table 3), which shows the relative time that the toxin and the scFv remain bound. The residence times of the scFv 10FG2 are the longest with the toxins Cll1 and Cn2, while for the remaining toxins, it is significantly shorter. Although this value is an important indicator of the neutralization capacity of a scFv, it was necessary to evaluate it in vivo in mice. Encouragingly, the neutralizing capacity of scFv 10FG2 against four toxins from three different venoms was noteworthy (Table 3). Toxins Cll1, Cll2, Cell9, and Ct1a were fully neutralized, despite differences in the *K_D_* and T_R_ values. These results contrast in a significant manner with the characteristics of the first set of scFvs described by our group (derived from scFv 3F) where the corresponding variants only become neutralizing when they reached affinities of the order of 1 × 10^−10^ M [20,25] and a time of residence (TR) of around 300 min. The incomplete neutralization of some venoms from which the evaluated toxins were isolated suggests the existence of other(s) component(s), such as the Cl13 toxin, which is present in *C. limpidus* venom, and is not neutralized by scFv 10FG2 (data not shown).

scFv 10FG2 was capable of fully neutralizing five venoms, as shown in the rescue assays where envenomed animals did not show any symptoms of intoxication at the end of the experiment (Table 4). It is important to bear in mind that in all of the recue assays, the intoxication progress was very fast, since three LD_50_ of fresh venom were applied. The strong symptoms of intoxication appeared between 10–20 min after the administration of the venom, contrasting with the 30–60 min required for these symptoms to appear when only one LD_50_ was injected. The relevance regarding these results is that some minutes after the administration of scFv 10FG2, the envenoming symptoms decreased notably, disappearing in a period of approximately 30 min, being evident that the animals recovered to a healthy state similar to that of unenvenomed mice. These results strongly support the possibility of simplifying the composition of antivenoms in a significant way, since a single antibody fragment can neutralize different toxins, as well as the whole venoms from several species of related scorpions. So far, demanding rescue tests implemented in mice have shown that the scFv format works as efficiently as the commercial antivenom (unpublished data), with the difference that the amount of antibody administered in scFv format is much lower. Several formats of recombinant antibodies have been generated depending on the different therapeutic purposes for which they have been developed [28]. In the case of the scorpion sting, given its immediate effect, an antidote with an equally rapid effect is required. From our point of view, despite some drawbacks that some authors have attributed to the single-chain format (rapid clarification and low stability), a rapid diffusion of antivenom to neutralize toxins can be critical, especially for children with advanced effects of envenoming. However, when reaching the point of preclinical testing, it is envisaged to test other formats and perform pharmacokinetic and pharmacodynamic tests to determine whether this is the best therapeutic format.

**Figure 3:**
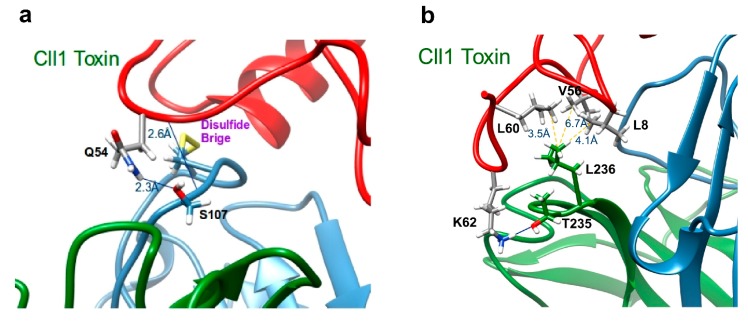
scFv 10FG2 differs with respect to its precursor (ER-1) in five residues. However, only three of them interact with the Cll1 toxin. These residues are shown. (**a**) S107 interacts with Q54 through its side chain and the main chain (see Table 5); (**b**) T235 interacts with K62 through its side chain, L236 shows hydrophobic interactions with L8, V56, and L60 (see Table 5). The Cll1 toxin is colored in red, while the scFv 10FG2 VH and VL domains are colored in blue and green, respectively.

When we analyzed the mutations present in scFv ER-1 that are associated with the broader cross-reactivity shown by scFv 10FG2 (A107S, I164S, A204G, L235T, and I236L (Table 2 and Figure 2), it was noticeable that only the changes to S107, T235, and L236 contacted the toxins through hydrogen bonds. Additionally, the residue L23established several hydrophobic interactions with residues 56 and 60 on the majority of toxins (Figure 3 and Table 4). For two other changes (S164 and G204) located in the corresponding frameworks, it is difficult to evaluate their contribution in the interaction, since they are situated far from the interface of the scFv 10FG2–toxin complex. However, SPR analyses indicated that S worked better than I. In the case of position 204, an analysis of the databases showed that G is a conserved residue in the FW3 of the light chain. This regress to the germline sequence represents an event that is similar to the G208 change that had been previously detected [22], and whose effect contributed to improving the interactions with toxins. These results indicate that in both positions (204 and 208), the presence of a glycine (consensus residues of the germlines) favored the interaction with the toxins.

The results of the analysis of the structural interactions by MD (Table 5) and the kinetics of molecular interactions in real time (Table 3) showed that the scFv 10FG2 bears a significantly higher affinity toward the Cll1 toxin, which correlates with the biggest number of interactions. Some interactions are absent in other molecular dynamics simulations of the complexes, even though the analyzed toxins have the same residues in the same positions as the toxins Cll1 and Cn2, which also correlate with the values of the *K_D_*, since scFv 10FG2 showed an affinity that was three times higher for the Cll1 toxin as compared to the Cn2 toxin. Likewise, the toxins recognized with lower affinity Css2, Cll2, and CeII9, whose *K_D_* values are in the same order of magnitude (see details in Table 3 and Table 5), showed a lower number of contacts during molecular dynamics (MD). This correlation was not observed with the Ct1a toxin, since scFv 10FG2 showed the lowest affinity despite the increase in the average number of hydrogen bonds observed in the MD simulations. We assume that the differences in the quality of the contacts (distance and duration) between the toxins and the scFv 10FG2 can explain the lower affinity.

The analyses of the interactions between scFv ER-1 (parental scFv in this work) [22] and scFv 10FG2 (matured) with the toxins, suggesting a structural adaptation or induced fit toward the different toxins in regard to the function of the changes that enabled improving the contacts in the interface. In this sense, the MD simulations of scFv variants 10FG2 and ER-1 showed that although residues I164 and G204 do not directly contact the toxins, they improve the binding energy at the interface (Table S1). The I164S change increased the hydrogen bonds on average from nine to 10.3 and enhanced the binding energy by −2.5 Kcal mol^−1^ for the Cll1 toxin; whereas for the Ct1a toxin, the average hydrogen bonds ranged from 7.2 to 10.4, and the binding energy was −2 Kcal mol^−1^ (Table 5 and Table S2). The change A204G did not result in any increase in the average of the hydrogen bonds during the interaction with the Cll1 toxin. The change was from 10.7 to 10.3, while the binding energy improved by −1.5 Kcal mol^−1^. In turn, for the Ct1a toxin, a slight increase in the average of hydrogen bonds from 10.0 to 10.4 was determined, but a significant increase in the binding energy of −1.9 Kcal mol^−1^ occurred. It is remarkable how these two changes could benefit the formation of the scFv 10FG2–toxin complexes. Despite a minimal increase in the number of hydrogen bonds at the interface, the interaction energy was improved similarly to that observed in the mutant A204G. Taken together, these data suggest that the quality of the contacts of scFv 10FG2 with a determined toxin can be modified by changing the particular residues that can affect the global structure of this antibody fragment.

## 4. Conclusions

The phenomenon of the cross-reactivity of antibodies is considered an unfortunate event in some cases. However, in instances in which it is necessary to neutralize several targets with similar characteristics, it is a great advantage, since a single molecule can be sufficient to achieve this objective. This is the case of traditional antivenoms, which are composed of antibodies that are capable of neutralizing the venom of several related species, such as the antivenoms against the scorpion sting and the bite of different terrestrial and marine snakes, among others. This capability can be explained in part by the polyclonal and polyvalent character of these antivenoms, but thanks to the development of key recombinant antibody fragments against scorpion toxins, we have been able to demonstrate that a single scFv can neutralize a significant number of toxins.

Through the implementation of different in vitro maturation strategies, it has been possible to evolve scFv C1, which in the beginning only recognized the Cn2 toxin contrasting with scFv 10FG2, which acquired the ability to recognize practically all the toxins of the venoms that have been studied in this work, and without losing the capacity for recognition to the initial antigen (Cn2 toxin). This result contrasts with the maturation process of the parental scFv 3F, where its main variants recognize and neutralize a smaller number of toxins, in addition to requiring greater affinities in order to be neutralizing [20,25].

Experimental results and structural analysis indicate that epitopes on the surface of the toxins with similar characteristics are present, which enables an important level of cross-reactivity. The scFv 10FG2 showed the ability to neutralize the toxins Cll1, Cll2, Ct1a, and CeII9, as well as the main toxins present in the venoms of *C. hirsutipalpus* (at least one), *C. infamatus* (Cii1), *C. noxius* (Cn2 and Cn3), *C. suffusus* (Css2 and Css4), *C. sp*. nov. “B” (two toxins), and C. sp. nov. “A” (one toxin) (unpublished data), which correspond to an estimated 13 different toxins. It is remarkable to have been able to generate a scFv with such a level of cross-neutralization, since we do not know other similar cases-verified in vivo tests in the case of scorpion toxins. The outlook is very encouraging, as we have verified the neutralizing capacity of this scFv against new toxins of species of dangerous scorpions that are just beginning to be characterized, which is thus expected to increase the number of toxins that are susceptible to being neutralized by 10FG2. Our main challenge is to neutralize the venom of all of the toxic scorpion species that are present in Mexico. For this reason, we are close to generating two new scFvs with a wide complementary cross-reactivity, which together with the scFv 10FG2 will enable achieving this demanding objective.

In summary, we have been able to develop a broadly specific scFv (capable of neutralizing five different venoms), which constitutes a significant simplification in the pharmaceutical composition of an innovative antivenom since, along with other few neutralizing scFvs, a cooperative effect in the neutralization of the entire venom of several species can be accomplished. All of these observations translate into a breakthrough toward the generation of a new recombinant antivenom of human origin against scorpion stings, and make avoiding the traditional antivenom production based on animal immunization possible.

## 5. Materials and Methods

### 5.1. In Vitro Maturation

#### 5.1.1. Library of scFv ER-1 by Site-Directed Saturation Mutagenesis

To perform saturation mutagenesis at positions L235 and I236, the oligos L3C1sat (5′-GG GAT AGC NNS NNS GGT TAT G-3′) and Cmyc (5′-TCA GAT CCT CTT CTG AGA ATG-3′) were used to obtain a megaprimer of approximately 150 bp. The expected amino acid diversity using this strategy is 1024. The megaprimer was purified by means of preparative agarose gel and used for a second PCR reaction in combination with the Dir oligo (5′-ATA CCT ATT GCC TAC GGC-3′) to obtain the full-size DNA fragment of approximately 900 pb for all of the scFv variants. The PCR product was purified by means of a preparative gel, which was digested with the restriction enzymes SfiI and NotI and ligated to the phagemid pSyn 2, which was already digested with the same enzymes [25]. The product of the ligation reaction was transformed into electrocompetent *E. coli* cells. The DNA isolated from several colonies were sequenced by the sequencing equipment model 3100 (Applied BioSystem: Foster City, CA, USA), and changes at the amino acid level were analyzed. The size of the library was 4 × 10^5^ transformants.

#### 5.1.2. Biopanning Process and Evaluation

The library of scFv ER-1 was displayed in phages and subjected to three rounds of screening against the toxins Cll2 and Ct1a independently. The biopanning process was carried out based on the protocol described in [25], with the following modifications. The toxin concentrations used were: 10 μg/mL, 1 μg/mL, and 0.5 μg/mL for Cll2, and 3 μg/mL, 1 μg/mL, 0.5 μg/mL for Ct1a. During the second and third rounds of screening, scFv ER-1 WT was used as the molecule competition in a ratio of 1:75, while toxin:scFv was used as the molecule competition for the second round, and a ratio of 1:80 was used for the third round. The identification of the improved variants was performed in ELISA plates using 3 μg/mL of each toxin, as described in Riaño et al. 2005 [25].

#### 5.1.3. Combinatorial Mutagenesis; Oligo Design and Construction of Variants of scFv 10F

Using the scFv 10F as a template, the combinatorial mutagenesis was made by the megaprimer strategy as described above using the oligos: C1H3A107S (5′-CCA CTC **GCT** GCA CAA TAG G-3′) to change A107S; C1Fw3 204A/G (5′-C CGA TTC TCT **GSC** TCC AAG-3′) so that position 204 could be mutated to A or G, and finally C1L1 I164S (5′-CT TGT TCT GGA **AGC** CGC TCC-3′) for the created I164S. The changes were made stepwise and cloned directly into the expression vector pSyn1. Once the constructs ware confirmed by sequence, the proteins of the scFvs were expressed and purified as described in [26]. The scFvs were purified by Ni2+-NTA affinity chromatography (Qiagen, Hilden, Germany), and eluted with 250 mm of imidazole. Finally, scFv preparations were purified by gel filtration chromatography on a SuperdexTM 75 column (Phamacia Biotech AB, Uppsala, Sweden). The protein concentrations of the monomers (a common characteristic in this scFv family) were determined spectrophotometrically at λ = 280 nm, using the molar extinction coefficient and the molecular weight of each scFv. For scFv, 10FG2 ε = 49,765 and MW = 28,527.34.

### 5.2. Comparison of the scFv Binding Properties by Surface Plasmon Resonance

The level of interaction of the scFv variants with toxins was determined using a biosensor that detects molecular interactions in real time (Biacore X, Uppsala, Sweden) at 25 °C. The Cll2, Ct1a, Cll1, Cn2, Css2, Css4, and CeII9 toxins were dissolved in 10 mM of 2-(N-morpholino) ethanesulfonic acid (pH 6) and immobilized on cell two of a CM5 sensor chip using the amino coupling kit, reaching binding levels of 300 RUs. Cell one in the sensor chip without antigen was used as a control.

The same concentrations of the scFv variants (100 nM) were used to evaluate the binding to each toxin. The samples were diluted in HBS-EP ( 0.01 M HEPES pH 7.4, 0.15 M NaCl, 3 mM EDTA, 0.005% v/v Surfactant P20 ) buffer (Biacore), and 100 µL of each scFv was injected over the chip at a flow rate of 50 mL min^−1^ with a delay of 500 s. The chip surfaces were regenerated with 10 mM of HCl. The sensorgrams were corrected by subtracting the values from the reference flow cell and comparing the results using BIA-evaluation software version 3.1.

### 5.3. Surface Plasmon Resonance Evaluations

The association and dissociation kinetic constants of the 10FG2 were determined as in [20] with some modifications. Serial dilutions of 10FG2 in HBS-EP buffer (Biacore) were injected into chips containing the immobilized Cll1, Cn2, Css2, Cll2, CeII9, or Ct1a toxins. Samples of 100 µL were injected over each chip at a flow rate of 50 µL min^−1^. The protein concentrations ranged from one nM to 50 nM for each assay. The delay phase lasted 600 seconds or 900 seconds. The kinetic constants were determined using the corresponding sensorgrams, which were corrected by subtracting the values from both the reference flow cell and the blank buffer injection. The Langmuir (1:1) model from BIA-evaluation software version 3.1 was used to determine the kinetic constants and the affinity constant (*K_D_* values are the ratios of association over dissociation constants). The time of residence (TR) of the scFvs on the different toxins was determined as the 1/koff value expressed in min.

### 5.4. Toxin Neutralization Assays

All of the in vivo neutralization assays were performed in accordance with the guidelines of the Bioethics Committee of the Institute of Biotechnology from the National Autonomous University of Mexico (IBT-UNAM—290 PDCPN 2014-01-246924). The beta-neurotoxins were purified from the scorpion venoms using previously described methodologies [8,29,30]. The in vivo neutralization tests were performed with the scFv 10FG2 against four toxins (Cll1, Cll2, CeII9, and Ct1a). A control group of eight CD1 mice weighing ~20 g was intraperitoneally injected with a lethal amount of each toxin (Table 3). In the experimental groups, the toxins were mixed with 10FG2 variant to prepare samples at a molar ratio of 1:5 toxin:scFv. The mixtures were preincubated for 30 min at room temperature (~25 °C) prior to injection. The alive animals were observed over a period of 48 hours, in order to guarantee that the symptoms of intoxication were totally eliminated. In those cases where unnecessary suffering was detected, the animals were slaughtered following the approved protocols of IBT-UNAM.

### 5.6. Neutralization Assays with Whole Fresh Scorpion Venoms

The in vivo neutralization tests were performed using the venom of scorpions recently collected to guarantee the highest toxicity of the venoms. The LD_50_ values that were used for each venom were determined previously intraperitoneally in CD1 mice [1]. Insoluble material was discarded, whereas the toxin-containing supernatant was recovered and spectrometrically quantified at λ = 280 nm, assuming that one unit of absorbance is equivalent of one mg/mL^−1^ of protein.

#### 5.6.1. Classical Protection

A control group of six female CD1 mice weighing ~20 g was intraperitoneally injected with venom amounts corresponding to two median lethal doses (LD_50_). For the neutralization assay, the amount corresponding to one LD_50_, two LD_50_, three LD_50_, four LD_50_, or five LD_50_ of each venom was mixed with a fixed amount of scFv 10FG2 (Table 4). The mixture of venom and scFv were preincubated at room temperature (~25 °C) for 30 min prior to their injection into the mice.

#### 5.6.2. Rescue Test

These experiments were designed for the determination of the ability of scFv 10FG2 alone and in combination with the scFv LR to rescue mice that were previously envenomed with a lethal amount of each venom (Table 4). A time span between 5–10 min was allowed to elapse before the mice were injected with different amounts of the antibodies representing 1:10 and 1:20 toxin:antibody molar ratios. The relative molar ratios were established assuming that 10% of each venom corresponded to toxic components.

### 5.7. Modeling and Structural Analyses of scFv 10FG2–Toxin Complexes

In order to establish the causes of cross-neutralization of scFv 10FG2 to the toxins studied in this work (see Figure 2 and Table 3), models of scFv 10FG2 complexed with every one of the six toxins of Figure 2A were assembled based on the scFv RU1-Cn2-scFv LR ternary complex structure [23]. Also, we modeled the structure of the linker amino acid sequence between the two variable domains of the single chain (from G125 to S139), because it was not defined. We submitted this protein sequence to secondary structure prediction to the YASPIN software [31], which predicted that the linker has no defined secondary structure; thus, it was structured as an extended chain.

The six scFv 10FG2–toxin complexes prepared were submitted to molecular dynamics (MD) simulation procedures. The complexes were first prepared with the System Builder application in the Maestro program, in which the complexes were soaked in a 15-Å buffered box of water with 0.15 M of NaCl and adjusted to minimize the volume. Using Viparr utility in the Desmond program, we settled the Charmm22star force field and the space water force field to all of the systems. Every system was then submitted to MD simulation with the Desmond program [32] with the following settings: a MD simulation time of 20 ns; trajectory recording intervals of 20 ps and five ps for energy recordings; an NVT (canonical ensemble class) at a temperature of 300 K; the Nose–Hoover thermostat method; and 100 ps of relaxation time. We use an integration time step of two ps and Coulombic radius cut off of nine Å (default values).

A sample of a thousand frames was extracted from the trajectories generated by MD simulations for the analysis of the contacts in the interphase between the scFV 10FG2 and the different toxins. From this sample, we took one of every 50 frames and submitted them to the PIC (Protein Interactions Calculator) software using default values [33] for their analysis. For the calculation of the binding free energy from MD trajectories, we analyzed the whole sample of frames (1000), and then, we averaged their values. For this purpose, we used a PERL programming language software designed ad hoc to evaluate every one of the sample’s frames, and then submitted them for analysis with the FoldX program [34].

## 6. Patents

The results contained in this manuscript were integrated into a requested patent in Mexico.

## Figures and Tables

**Table 1:** Primary structure and relative abundance of the principal toxic components from some scorpion venoms.

Species	Toxin	Sequence	%
*C. noxius*	Cn2	KEGYLVDKNTG**C**KYE**C**LKLGDNDY**C**LRE**C**KQQYGKGAGGY**C**YAFA**C**W**C**THLYEQAIVWPLPNKR**C**S	6.8
*C. limpidus*	Cll1	KEGYIVNLSTG**C**KYE**C**YKLGDNDY**C**LRE**C**KQQYGKGAGGY**C**YAFG**C**W**C**THLYEQAVVWPLPKKT**C**T	1.5
	Cll2	KEGYLVNHSTG**C**KYE**C**FKLGDNDY**C**LRE**C**KQQYGKGAGGY**C**YAFG**C**W**C**NHLYEQAVVWPLPKKT**C**N	3.5
	Cl13	KEGYLVDYHTG**C**KYT**C**AKLGDNDY**C**VRE**C**RLRYYQSAHGY**C**YAFA**C**W**C**THLYEQAVVRPLPNKR**C**R	2.1
*C. suffussus*	Css2	KEGYLVSKSTG**C**KYE**C**LKLGDNDY**C**LRE**C**KQQYGKSSGGY**C**YAFA**C**W**C**THLYEQAVVWPLPNKT**C**N	2.8
*C. tecomanus*	Ct1a	KEGYLVNHSTG**C**KYE**C**FKLGDNDY**C**LRE**C**RQQYGKGAGGY**C**YAFG**C**W**C**THLYEQAVVWPLPKKT**C**N	1.8
*C. elegans*	Cell9	KEGYLVNHSTG**C**KYE**C**FKLGDNDY**C**LRE**C**RQKYGKGAGGY**C**YAFG**C**W**C**THLYEQAVVWPLPKKT**C**N	2.9
*C. infamatus*	Cii1	KEGYLVNHSTG**C**KYE**C**YKLGDNDY**C**LRE**C**KQQYGKGAGGY**C**YAFG**C**W**C**THLYEQAVVWPLPKKT**C**N	ND

Pattern of disulfide bridges: C12–C65, C16–C41, C25–C45, and C29–C48. Cysteine residues are indicated in bold characters. Medically important venoms contain between one and three different major toxic components. The symbol % indicates the percentage of relative abundance in the venom. Cn: *Centruroides noxius*; Css: *Centruroides suffusus*; Ct: *Centruroides tecomanus*; Cii: *Centruroides infamatus*; Cll: *Centruroides limpidus*; CeII9: *Centruroides elegans*

**Table 2:** Maturation process.

scFv	VH						VL					REF.
	CDR2			CDR3			FW1	FW3		CDR3		
**C1**	54 S	56 D	57 N	105 M	107 A	110 Y	164 S	204 A	208 D	235 L	236 I	[25]
RU1		G	G	L		H						[23]
ER-1	G	G	S	L		T	I		G			[22]
10F	G	G	S	L		T	I		G	T	L	^α^
**10F1**	**G**	**G**	**S**	**L**		**T**	**I**	**G**	**G**	**T**	**L**	^α^
**10F2**	**G**	**G**	**S**	**L**		**N**		**G**	**G**	**T**	**L**	^α^
**10F3**	**G**	**G**	**S**	**L**		**N**			**G**	**T**	**L**	^α^
**10F4**	**G**	**G**	**S**	**L**	**S**	**N**		**G**	**G**	**T**	**L**	^α^
**10F5**	**G**	**G**	**S**	**L**	**S**	**T**		**G**	**G**	**T**	**L**	^α^
**10F6**	**G**	**G**	**S**	**L**	**S**	**N**			**G**	**T**	**L**	^α^

^α^ ER-1 variants generated in this work; mutation positions are shown. VH corresponds to the variable domain of antibody heavy chain and VL is the corresponding variable domain of a light chain. CDR stands for complementarity-determining region and FW means framework. scFvs indicated in bold correspond to variants derived from scFv 10F.

**Table 3:** Characterization of scFv 10FG2.

**(A)**					
**TOXIN**	***k_on_*** **(1/Ms) ×** **10^5^**	***k_off_*** **(1/s) ×** **10^−5^**	***K_D_*** **(M) ×** **10^−9^**	**T_R_ (min)**	***K_D_* (M) scFv × 10^−9^ ER**
Cll1	2.65 ± 0.65	6.3 ± 1.3	0.23 ± 0.005	264.5	1.3
Cn2	1.50 ± 0.30	10.2 ± 0.8	0.70 ± 0.075	163.4	1.0
Css2	1.95 ± 0.05	55.0 ± 5.0	2.75 ± 0.25	30.0	ND
Cll2	2.20 ± 0.50	90.0 ± 10.0	4.20 ± 0.40	18.5	5.8
CeII9	1.50 ± 0.10	92.5 ± 4.5	6.10 ± 0.10	18.0	ND
Ct1a	1.70 ± 0.81	100.0 ± 0.01	8.00 ± 4.00	16.7	29.0
**(B)**					
**Toxin**	**µg/20 g Mouse**	**Alive/Total**			
		**Control**		**Pre-Incubated Mix**	
Cll1	1.7	1/8		8/8	
Cll2	1.5	0/8		8/8	
CeII9	3.0	0/8		8/8	
Ct1a	2.0	0/8		8/8	

(A) Kinetic constants of scFv 10FG2 against several toxins. Biosensor assays were performed at 25 °C at a flow rate 50 µL min^−1^. The constants were calculated using the Langmuir (1:1) model in the Bia-evaluation 3.1 software. In the right column, *K_D_* values for the scFv ER-1 [22]. ND: non determined. T_R_: time of residence. (B) Neutralization tests of scFv 10FG2 against Cll1, Cll2, CeII9, and Ct1a toxins. Mice were intraperitoneally injected with the toxin (controls) or with a preincubated mixture of toxin and antibody (experimental) at a molar ratio of 1:5 (toxin: scFv).

**Table 4:** Venoms neutralization tests.

**(A)**							
**Scorpion Venom**	**Control**		**Alive/Total** **LD_50_ Number and Molar Ratios**				
	**2 LD_50_**	**scFv µg/mouse**	**1 LD_50_** **1:10**	**2 LD_50_** **1:5**	**3 LD_50_** **1:3.3**	**4 LD_50_** **1:2.5**	**5 LD_50_** **1:2**
***C. hirsutipalpus***	0/6	44.5	6/6	6/6	6/6	3/6	ND
***C. infamatus***	0/6	36.4	6/6	6/6	6/6	6/6	6/6
***C.* sp nov. B**	0/6	73.0	6/6	6/6	5/6	N/D	ND
***C. suffusus***	0/6	33.2	6/6	6/6	6/6	6/6	ND
***C. noxius***	0/6	9.5	6/6	6/6	6/6	6/6	ND
***C.* sp nov. A**	0/6	49.4	6/6	1/6	ND	ND	ND
**(B)**							
**Venom**	**Alive/Total**		**Molar Ratio**		**µg of Venom/µg of scFv/20 g Mouse**		
***C. hirsutipalpus***	6/6		1:10		35.1/133.4		
***C. infamatus***	6/6		1:10		28.8/109.4		
***C.*** **sp nov. B**	6/6		1:10		57.6/218.9		
***C. noxius***	5/6		1:10		7.5/28.5		
***C. suffusus***	5/6		1:10		26.5/99.8		
***C. noxius***	6/6		1:20		7.5/57.0		
***C. suffusus***	6/6		1:20		26.5/199.6		

(A) Neutralization challenge of scFv 10FG2 with several LD_50_ of whole fresh soluble venom from six toxic *Centruroides* species. Controls corresponded to two LD_50_ of each venom freshly obtained. Unless otherwise indicated, mixtures were prepared using a fixed amount of antibody with a different number of LD_50_ and preincubated for 30 min at 37 °C. The relative molar ratios were calculated assuming that toxins represent 10% of each venom. sp. nov: *species nova* (B) Rescue assays. Mice were previously intoxicated with three LD_50_ of venom. In all of the cases, in the control group, three LD_50_ of each venom were administered, and none of the mice survived.

**Table 5:** Interactions at the interface between scFv 10FG2 and different toxins through molecular dynamics (MD) simulations.

Residues of scFv 10FG2	Residues of Cll1 Toxin	Residues of Cn2 Toxin	Residues of Css2 Toxin	Residues of Cll2 Toxin	Residues of Ct1a Toxin	Residues of Cell9 Toxin
Y59	L8					
L105	I5		L5		L5	
L105	V6	V6	V6	V6	V6	V6
L105	L8					
L105	Y33	Y33	Y33	Y33	Y33	Y33
L105	A55					
L105	V56	I56	V56	V56	V56	V56
W231	V56	I56	V56	V56		V56
L236	L8					
L236	V56	I56	V56		V56	V56
L236	L60	L60	L60			L60
Hydrogen Bonds						
**S31(O)**	K35(NZ)	K35(NZ)	K35(NZ)	K35(NZ)	K35(NZ)	K35(NZ)
S31(OH)		K35(NZ)	K35(NZ)	K35(NZ)	K35(NZ)	
S52(OH)	**Q31(O)**	**Q31(O)**	**Q31(O)**			
S52(OH)	Q31(OE1)	Q31(OE1)	Q31(OE1)	Q31(OE1)	Q31(OE1)	Q31(OE1)
**G54(N)**	Q31(OE1)	Q31(OE1)	Q31(OE1)	Q31(OE1)	Q31(OE1)	Q31(OE1)
**G55(N)**	Q31(OE1)					
**G56(N)**	Q31(OE1)	Q31(OE1)	Q31(OE1)	Q31(OE1)	Q31(OE1)	Q31(OE1)
Y53(OH)	**K30(O)**		**K30(O)**	**K30(O)**	**R30(O)**	**R30(O)**
S57(OH)	Q32(OE1)	Q32(OE1)			K32(NZ)	Q32(OE1)
S57(OH)			**Q31(O)**	**Q31(O)**		
Y59(OH)	Q32(OE1)	Q32(OE1)	Q32(OE1)	Q32(OE1)		Q32(OE1)
Y59(OH)	Q32(NE2)	Q32(NE2)			K32(NZ)	Q32(NE2)
Y59(OH)		**D7(O)**				
Y59(OH)	N7(OD1)					N7(OD1)
Y59(OH)						N7(ND2)
Y59(OH)	**L8(N)**	**K8(N)**	**K8(N)**	**H8(N)**	**H8(N)**	**H8(N)**
**Y60(O)**		K8(NZ)	K8(NZ)			
D62(OD1)		K8(NZ)				
D62(OD1)				K63(NZ)		
K65(NZ)	**S9(O)**	N9(OD1)		**S9(O)**		**S9(O)**
K65(NZ)						S9(OH)
R101(NH2)	Q54(OE1)	Q54(OE1)	Q54(OE1)	Q54(OE1)	Q54(OE1)	Q54(OE1)
R101(NH1)	Q54(NE2)		Q54(NE2)	Q54(OE1)		Q54(OE1)
D102(OD1)	Y52(OH)	Y52(OH)	Y52(OH)	Y52(OH)	Y52(OH)	Y52(OH)
D102(OD2)	K35(NZ)	K35(NZ)	K35(NZ)	K35(NZ)	K35(NZ)	K35(NZ)
D102(OD2)	**K35(N)**	**K35(N)**	**K35(N)**	**K35(N)**	**K35(N)**	**K35(N)**
**L104(N)**	**Q32(O)**	**Q32(O)**	**Q32(O)**	**Q32(O)**	**K32(O)**	**Q32(O)**
**L105(N)**	**Q32(O)**	**Q32(O)**	**Q32(O)**	**Q32(O)**	**K32(O)**	**Q32(O)**
**L105(O)**	**V56(N)**	**L56(N)**	**V56(N)**	**V56(N)**	**V56(N)**	**V56(N)**
**S107(N)**	**Q54(O)**	**Q54(O)**	**Q54(O)**	**Q54(O)**	**Q54(O)**	**Q54(O)**
S107(OH)	**Q54(O)**		**Q54(O)**	**Q54(O)**	**Q54(O)**	**Q54(O)**
S107(OH)	Q54(NE2)	Q54(NE2)			Q54(NE2)	Q54(NE2)
D108(OD2)	Q54(NE2)	Q54(NE2)	Q54(NE2)		Q54(NE2)	Q54(NE2)
D108(OD1)						Q54(NE2)
**S165(O)**					K62(NZ)	
N171(OD1)						Q54(NE2)
T172(OH)		Q54(OE1)				
**T172(N)**						Q54(OE1)
AS190(OH)					Q54(NE2)	
**D233(O)**	K62(NZ)	N62(ND2)	N62(OD1)			K62(NZ)
D233(OD2)			N62(ND2)			K62(NZ)
S234(OH)	K62(NZ)	N62(NZ)		K62(NZ)		K62(NZ)
**T235(O)**		K8(NZ)	K8(NZ)			
T235(OH)	K62(NZ)	N62(ND2)	K62(NZ)	K62(NZ)		
T235(OH)					K63(NZ)	
**L236(O)**		K8(NZ)	K8(NZ)			
**G237(O)**			K8(NZ)			
Ionic Interactions within Six Angstroms						
D102	K35					
Cation–Pi Interactions within Six Angstroms						
Y53	K35					
R101	Y52					
Number contacts ^α^	42	36	34	27	29	36
Hydrogen Bonds ^δ^	10.3	10.8	10.3	10.4	9.1	10.4

Bold types denote main-chain atoms involved in contacts. Underlined types denote toxin residue positions in which there are differences among toxins (see Figure 2A). ^α^ Total number of contacts along MD simulations; ^δ^ Hydrogens bonds average of MD simulations.

## Supplementary Materials

The following are available online at http://www.mdpi.com/2072-6651/11/1/32/s1, Table S1: Comparative ΔG° of dissociation of scFvs 10FG2 and ER-1, Table S2: ΔG° of binding and Hydrogen Bonds average of 10FG2 and its revertant mutants with Cll1 and Ct1a toxins.
