# Supplementary material for: Generation of a Broadly Cross-Neutralizing Antibody Fragment against Several Mexican Scorpion Venoms
Source: Toxins (Basel). 2019 Jan 10;11(1):32. doi: 10.3390/toxins11010032 (PMC6356842; doi:10.3390/toxins11010032)
Supplement: Supplementary file 1 [file toxins-11-00032-s001.pdf]

Supplementary Materials: Generation of a Broadly Cross-Neutralizing Antibody Fragment against Several Mexican Scorpion Venoms

Lidia Riaño-Umbarila, Ilse V. Gómez-Ramírez, Luis M. Ledezma-Candanoza, Timoteo Olamendi-Portugal, Everardo Remi Rodríguez-Rodríguez, Guillermo Fernández-Taboada, Lourival D. Possani and Baltazar Becerril

Table S1. Comparative ΔG° of dissociation of scFvs 10FG2 and ER-1.

Toxin	scFv 10FG2 ΔG° of dissociation in Kcal·mol^{-1}	ScFv ER-1 ΔG° of dissociation in Kcal·mol^{-1}
Cll1	13.1	12.1
Cn2	12.5	12.3
Cll2	11.4	11.2
Ct1a	11.0	10.3

Table S2. ΔG° of binding and hydrogen bonds average of 10FG2 and its revertant mutants with Cll1 and Ct1a toxins.

	scFv 10FG2 ΔG°$_{bind}$ (Kcal·mol^{-1})	H.B.	scFv 10FG2 S107A ΔG°$_{bind}$ (Kcal·mol^{-1})	H.B.	scFv 10FG2 S164I ΔG°$_{bind}$ (Kcal·mol^{-1})	H.B.	scFv 10FG2 G204A ΔG°$_{bind}$ (Kcal·mol^{-1})	H.B.	scFv10FG2 T235L/I236L ΔG°$_{bind}$ (Kcal·mol^{-1})	H.B.
Cll1	-8.3	10.0	-8.4	9.7	-5.9	9.0	-6.9	10.7	-8.1	9.7
Ct1a	-6.0	11.0	-5.5	10.1	-3.7	7.2	-3.8	10.0	-7.9	8.9

ΔG°$_{bind}$ represents the Gibbs free energy of binding calculated from MD simulations. H.B. represents the hydrogen bonds average along MD Simulations.
